# Effects of caregiver dementia training in caregiver‐patient dyads: A randomized controlled study

**DOI:** 10.1002/gps.5378

**Published:** 2020-07-22

**Authors:** Elizabeth G. Birkenhäger‐Gillesse, Wilco P. Achterberg, Sarah I.M. Janus, Boudewijn J. Kollen, Sytse U. Zuidema

**Affiliations:** ^1^ Department of General Practice and Elderly Care Medicine University of Groningen, University Medical Center Groningen Groningen The Netherlands; ^2^ Laurens Care Centers, Division Long Stay Rotterdam The Netherlands; ^3^ Department of Public Health and Primary Care Leiden University Medical Center Leiden The Netherlands

**Keywords:** caregiver, dementia, psychosocial intervention, training

## Abstract

**Objectives:**

Caregivers for people with dementia (PWD) have reported needing emotional and social support, improved coping strategies, and better information about the illness and available support services. In this study, we aimed to determine the effectiveness of an Australian multicomponent community‐based training program that we adapted and implemented in a non‐medical Dutch health care setting.

**Methods and design:**

A randomized controlled trial was performed: 142 dyads of cohabiting caregivers and PwD were randomized to control (care as usual) or intervention (training program) groups and outcomes were compared. Programs lasted 1 week, comprised 14 sessions, and were delivered by specialist staff. We included 16 groups of two to six caregivers. The primary outcome was care‐related quality of life (CarerQol‐7D) at 3 months. The main secondary outcomes for caregivers were self‐rated burden, health and mood symptoms, and for PwD were neuropsychiatric symptoms, quality of life, and agitation.

**Results:**

No significant difference was observed for the primary outcome. However, caregivers experienced fewer role limitations due to physical function (adjusted mean difference, 13.04; 95% confidence interval [95%CI], 3.15‐22.93), emotional function (13.52; 95%CI, 3.76‐23.28), and pain reduction (9.43; 95%CI, 1.00‐17.86). Positive outcomes identified by qualitative analysis included better acceptance and coping and improved knowledge of dementia and available community services and facilities.

**Conclusion:**

Quantitative analysis showed that the multicomponent course did not affect care‐related quality of life but did have a positive effect on experienced role limitations and pain. Qualitative analysis showed that the course met the needs of participating dyads.

**Key points**

- A multicomponent caregiver training did not affect care related quality of life of caregivers who live with a person with dementia
- Caregivers who live with a person with dementia experienced less role limitations and less pain as a result of a multicomponent caregiver training.
- Caregivers who live with a person with dementia reported that the course met a wide variety of their needs depending on their individual situation.

## 1 INTRODUCTION

Worldwide about 50 million people suffer from dementia,^1^ with many of these preferring to live at home for as long as possible to maintain their social network and quality of life (QoL).^2^ In the Netherlands it is estimated that 70% of people with dementia (PwD) live at home and receive care by informal caregivers, and that 35% of those caregivers are spouses.^3^ Caring for a PwD can result in poor mental health, with females disproportionally affected.^4^, ^5^, ^6^, ^7^, ^8^ Caregivers also have high and persistent rates of burden that is negatively correlated with QoL.^9^ This high burden is related to the poorer cognition, neuropsychiatric symptoms, and functional impairment of the PwD and may lead to nursing home placement.^2^, ^10^, ^11^

Studies looking at the needs of caregivers have shown that emotional and social support, improving coping strategies, and providing information about the illness and available support services can help reduce caregiver burden. Interventions based on these needs should therefore be central to any efforts to improve the health of caregivers.^12^, ^13^ Single component psychosocial and behavioral interventions yield small but significant effects on caregiver burden, depression, QoL, stress, and sense of competence.^14^, ^15^ However, multicomponent interventions have also been shown to yield small but significant effects on burden, depression, health, and social support for caregivers.^16^, ^17^, ^18^ Reviews of both types of intervention strategy indicate that support groups should address educational and therapeutic components while targeting cognitive appraisals and coping styles in the caregiver.^16^, ^17^, ^18^, ^19^

In Australia, a residential multicomponent training program was developed for caregivers living with PwD. This program included psychological and educational themes and was delivered in informally structured group sessions with educational elements, group work, modeling, and role‐play.^20^, ^21^ The results of a randomized controlled trial comparing waiting and control groups (respite care) showed that the training program effectively delayed nursing home admission, reduced mortality, reduced psychological morbidity, and lowered care costs.^20^, ^22^, ^23^, ^24^ An extension study using a pre‐post design in another setting produced comparable results.^21^, ^25^ However, the original Australian trial was performed more than 30 years ago and in a different healthcare system to that in the Netherlands.

In this study, we adapted the Australian residential multicomponent training program, seeking to deliver it in a non‐medical setting in the Netherlands. Our aim was to determine its effects on care‐related QoL in caregivers (primary outcome) and on other relevant secondary outcomes in caregivers and PwD.

## 2 METHODS

### 2.1 Ethics

The study was submitted for approval to the Human Research Ethics Committee of the University of Groningen, the Netherlands, before starting. The committee concluded that no assessment was needed based on relevant Dutch law concerning scientific research in humans, and the study was also carried out in accordance with the ethical standards of the Declaration of Helsinki (1964, and subsequent revisions). Written informed consent was obtained from all participating caregivers, and if possible, from the PwD. The trial has been registered at the Dutch Trial Register; Trial ID, NTR5775.

### 2.2 Design and participants

In this randomized controlled trial, we randomly assigned participant dyads (a caregiver and a PwD living together) to intervention or control groups. Randomization was performed by block randomization by the research assistant who was blind to the pre‐fixed treatment allocations and to the block size. Participants were recruited to the “More at Home with Dementia” study (in Dutch, *Beter Thuis met Dementie*), either by professional referral or by self‐referral. To improve recruitment, we developed a logo, a website (http://beterthuismetdementie.laurens.nl/), a short promotional film, a brochure, a Facebook page, a local newspaper advertisement, and regular newsletters. Participant dyads in the intervention group took part in the study training program, while those in the control group received care as usual. Quantitative data were collected at baseline and at 3 and 6 months after the intervention, while qualitative data on the intervention's effects were collected after 3 and 6 months. The full trial protocol has been published elsewhere.^26^

### 2.3 Intervention

Each intervention lasted 5 days and took place in holiday accommodation. In total, 16 groups consisting of two to six participant dyads received the intervention between May 2016 and March 2018. The caregivers attended 14 psycho‐educational group sessions on all relevant emotional, relational, practical, financial and social changes that come with living with someone with dementia. These were delivered in an informal setting by a psychologist, a physiotherapist, an occupational therapist, an elderly care physician, a speech therapist, a dietician, and a social worker. The sessions included psycho‐educational elements, group work, modeling, and role‐play. The use of a facilitator's guide guaranteed the workshops included the same content when staff changed. A syllabus was made of all the theoretical content from the workshops and was provided as a plain language reference book to all participants. The program for the PwD comprised general pleasant activities and sessions focused on coping with the handicaps that come with dementia. When appropriate, some of the workshops were given to the caregivers and PwD together. Apart from the intervention participants continued to receive care as usual. A more in‐depth description has been published elsewhere.^26^

### 2.4 Control group

Participants assigned to the control group received care as usual. This comprised support being offered to the caregiver either by a dementia case manager, by other support groups, or by day care centers offering respite care, or any combination of these. However, there were major regional differences in the availability of these services and facilities.

### 2.5 Measurements

#### 2.5.1 Primary outcome in caregivers

• Care‐Related Quality of Life‐7 dimensions (CarerQol‐7D). This measure scores seven items on care‐related satisfaction, relationship problems, mental health, time management, financial problems, social support, and physical health. All items are scored on a three‐point scale.^27^ The scores are then transformed to represent a utility score or tariff between 0 (worst informal care situation) and 100 (best informal care situation) by adding the relative item weights.^28^

#### 2.5.2 Secondary outcomes in caregivers

- CarerQol—visual analog scale (VAS). For the VAS, 0 equaled “completely unhappy” and 10 equaled “completely happy.”^28^
- Self‐Rated Burden Scale—VAS. A self‐report measure of burden experienced in the caregiver role: 0 equaled “no burden” and 10 equaled “too much burden.”^27^, ^29^
- RAND‐36/short form (SF). This was used to measure experienced health or health‐related QoL. The survey includes eight separate scales concerning physical function, role limitations due to physical health problems, bodily pain, general health perception, vitality, social function, role limitations due to emotional problems, and general mental health.^30^
- EuroQol‐5 Dimensions (EQ‐5D‐3L). A health instrument that assesses QoL on five dimensions: mobility, self‐care, activities, pain and discomfort, and anxiety and depressed mood.^31^, ^32^, ^33^
- Center of Epidemiologic Studies‐Depression (CES‐D). This instrument screens for depressive symptoms across 20 items.^34^
- Hospital Anxiety and Depression Scale‐Anxiety Subscale (HADS‐A). The HADS is a well‐known 14‐item scale that generates ordinal data, with seven items that determine anxiety levels.^35^
- Perseverance Time. This was defined as the length of time caregivers believed they could persevere in their current situation if it remained unchanged.^36^, ^37^

#### 2.5.3 Secondary outcomes in persons with dementia (informant‐rated)

- The 12‐item neuropsychiatric Inventory (NPI) was used to measure the frequency and severity of neuropsychiatric symptoms in PwD.^38^
- Functional status. We used an adapted version of the Katz Index of Independence of Activities for Daily Living and instrumental activities of daily living, plus a question on mobility.^39^
- EuroQol‐5 Dimensions + Cognition (EQ‐5D + C). A question about cognitive function was added to the EQ‐5D‐3L to improve relevance to the PwD.^31^, ^32^, ^33^
- The Dementia Quality of Life Instrument (DQI). This tool covers five health domains: memory, orientation, dependency, social activities, and mood, and is self‐rated when possible.^40^, ^41^
- The Cohen‐Mansfield Agitation Inventory‐Community (CMAI‐C) scale was used to measure the frequency of agitated behavior in PwD.^42^
- The Reisberg Global Deterioration Scale (GDS). This scale was used to classify PwD based on the relative severity of their cognitive impairment and their functional status.^43^

#### 2.5.4 Secondary outcomes in both caregivers and persons with dementia

- Psychotropic drug use. Coding was done according to the Anatomical Therapeutic Chemical classification system of the World Health Organization. Medication was categorized as follows: (a) all psychotropics, except antidementia drugs; (b) antipsychotics; (c) antidepressants; and (d) hypnotics and anxiolytics. Only medication prescribed for daily use was included.

### 2.6 Qualitative data

At meetings after 3 and 6 months, caregivers were asked to expand on three questions: (a) which workshops were most helpful, and what knowledge had they actually put into practice; (b) what difference the intervention had made to their life as a caregiver; and (c) how they had experienced the intervention week, including any areas they saw for improvement. These meetings were audio recorded and summarized by two independent research assistants.

### 2.7 Statistical analysis

#### 2.7.1 Sample size

Including 144 couples divided equally among the intervention and control groups was shown to offer enough power (0.8) to demonstrate an effect size of 0.5 at a significance level of 0.05, allowing for an anticipated attrition rate of about 15%. This estimated sample size is similar to that used by Brodaty et al in their largest group.^22^

#### 2.7.2 Analysis

For the primary and secondary outcomes, linear regression modeling was used to test the response variable for differences between the intervention and control groups adjusted for baseline outcome scores (both unadjusted and adjusted estimates are reported). Outcomes are reported as the adjusted mean differences (aMD) and their 95% confidence intervals (95%CI). Regression analyses were conducted on a modified intention‐to‐treat basis and checked for whether they met the required assumptions. Subgroup analyses were performed for the primary outcome by sex, age, and education level. Age was dichotomized based on the mean age of caregivers, and education level was dichotomized by attainment of at least upper secondary education. However, due to high dropout rates at 6 months, these data were not used for further analyses. Analysis was conducted to identify patterns in the missing values. Due to some of the caregivers having to complete the CMAI and NPI questionnaires without assistance, up to four values were missing per question in 12% and 24% of cases, respectively. Assuming that these were missing at random, we used multiple imputation techniques (20 times) to estimate and replace the values missing at baseline and at 3 months.

## 3 RESULTS

### 3.1 Participants

**FIGURE 1:**
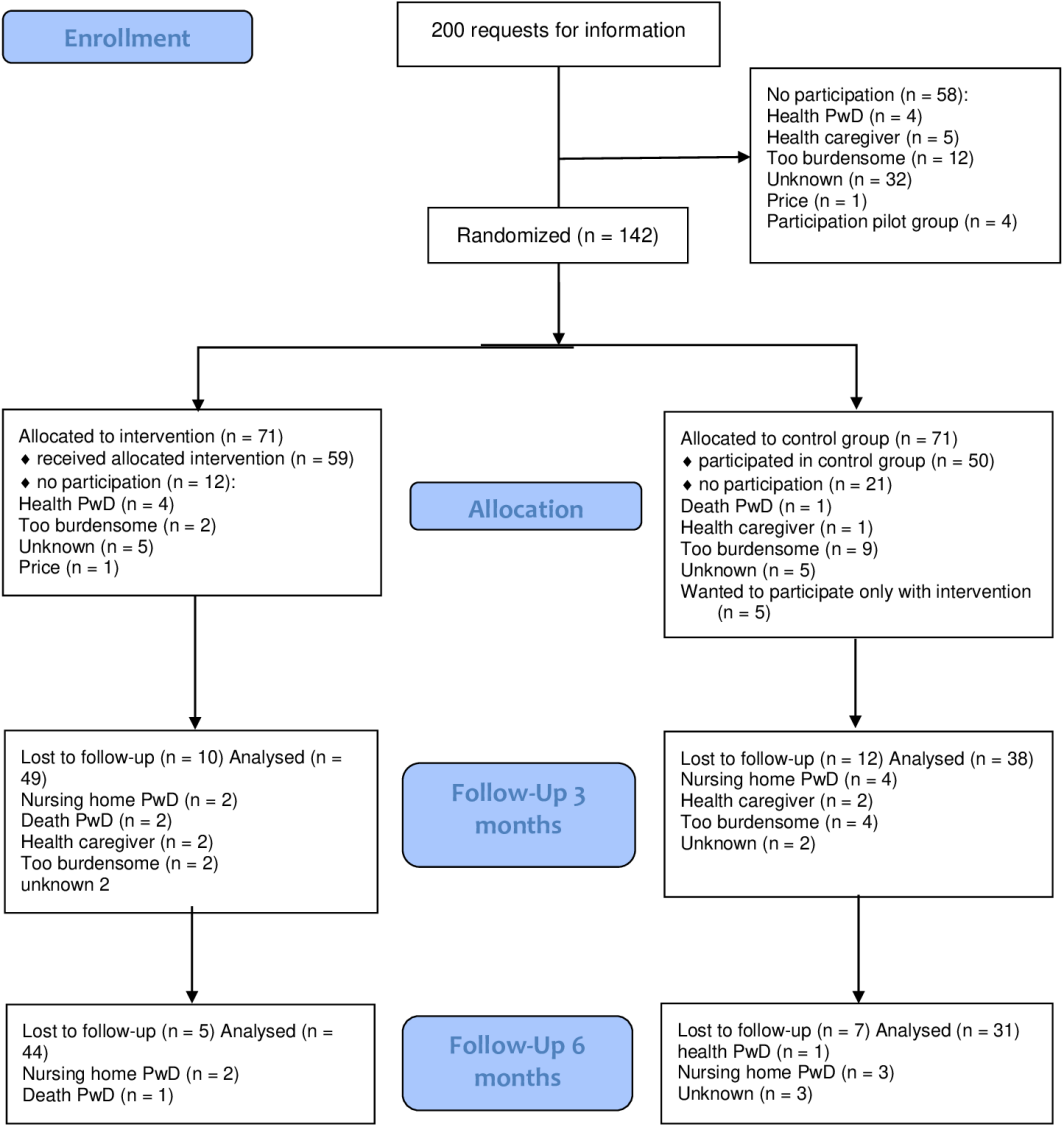
Participation flowchart. Data are for those after request for information and include the reasons for dropout [Colour figure can be viewed at wileyonlinelibrary.com]

In total, 200 people contacted us for information on the project. Of these, 58 opted not to participate, mostly because of health reasons or the perceived burden of the intervention, resulting in 142 participants being randomized to the study groups. However, 12 couples in the intervention group and 21 in the control group dropped out before the study began, leaving 59 and 50 eligible for baseline analysis, respectively (Figure 1). Reasons for dropout varied by group: 9 and 2 couples resigned from the control and intervention groups, respectively, because they expected the survey to be overly burdensome, and another five participants in the control group resigned because they had a strong preference to be in the intervention group. After the intervention and baseline measures, 10 participants in the intervention and 12 participants in the control group dropped out before 3 months; another 5 and 7, respectively, dropped out before 6 months. Some questionnaires were partially completed or not completed at all, causing additional missing data.

### 3.2 Baseline characteristics

The baseline characteristics are presented in Table 1. Caregivers in the intervention and control groups had mean ages of 72.5 and 73.2 years, respectively; the corresponding ages of the PwD were 76.3 and 77.6 years. Caregivers were about 4 years younger than PwD and most were women (75%). Almost all PwD had moderate to moderately severe dementia and were born in the Netherlands.

**TABLE 1:** Baseline characteristics

	Intervention	Control
CAREGIVER, N	59	49
Age in years, mean (SD)	72.5 (8.3)	73.2 (7.1)
Women, %	76.3	74.0
PERSON WITH DEMENTIA, N	59	50
Mean age in years (SD)	76.3 (6.7)	77.6 (7.3)
Born in the Netherlands, %	100	98
Upper secondary education or more, %	72.4	70.5
Number, N	53	43
GDS, mean (SD)^a^	4.6 (0.79)	4.4 (0.79)

^a^ GDS: Reisberg Global Deterioration Scale, range 1 to 7, higher scores indicating more severe dementia.

Abbreviation: SD, standard deviation.

### 3.3 Primary outcome

Table 2 shows the primary and secondary outcomes for both the caregiver and PwD groups. At 3 months, the intervention group did not have a significantly higher QoL according to their score on the Carer Qol‐7D (aMD, 5.12; 95%CI −1.09 to 11.33).

**TABLE 2:** Primary and secondary outcomes after 3 months

	Range	B (95%CI) crude	*P* value	B (95%CI) adjusted for baseline	*P* value
CAREGIVER					
Carer Qol‐7D	0‐100^a^	6.43 (−1.83, 14.69)	.13	5.12 (−1.09, 11.33)	.11
Carer Qol VAS	0–10 ^a^	0.006 (−0.71, 7.21)	.99	0.16 (−0.38, 0.70)	.56
Self‐rated Burden Scale	0–10 ^b^	−0.40 (−1.32, 0.52)	.39	0.12 (−0.53, 0.78)	.71
RAND 36 Short Form					
*Physical functioning*	0–100 ^c^	1.54 (−8.85, 11.93)	.77	1.86 (−5.4, 9.13)	.61
*Role limitations due to physical functioning*	0–100 ^c^	9.58 (−3.07, 22.23)	.14	13.04 (3.15, 22.93)	.01
*Pain*	0–100 ^c^	7.34 (−2.85, 17.54)	.16	9.43 (1.00, 17.86)	.03
*General health perception*	0–100 ^c^	5.14 (−3.72, 13.99)	.25	4.58 (−2.52, 11.68)	.20
*Mental health*	0–100 ^c^	2.88 (6.16, 11.92)	.53	2.57 (−6.69, 11.83)	.58
*Health change*	0–100 ^c^	0.90 (−6.27, 8.07)	.80	1.52 (−5.34, 8.39)	.66
*Social functioning*	0–100 ^c^	5.46 (−4.65 15.56)	.29	5.44 (−3.08, 13.97)	.21
*Role limitations due to emotional functioning*	0–100 ^c^	10.28 (−2.20 22.75)	.11	13.52 (3.76, 23.28)	<.01
*Vitality*	0–100 ^c^	2.17 (−6.59 10.93)	.62	2.76 (−3.08, 8.59)	.35
EQ 5D‐3L	max. 1 ^a^	0.07 (−0.013 0.153)	.10	0.06 (−0.02, 0.13)	.12
CES‐D	max. 60 ^d^	−1.33 (−5.69, 3.02)	.54	−2.45 (−5.67, 0.77)	.13
HADS‐A	0–21 ^e^	0.67 (−0.33, 1.67)	.19	0.46 (−0.21, 1.13)	.18
Perseverance time	1–6 ^f^	0.14 (−0.30, 0.59)	.53	0.13 (−0.27, 0.52)	.53
PERSON WITH DEMENTIA					
KATZ 15	0–15 ^g^	−1.50 (−3.13, 0.13)	.07	−0.54 (−1.30, 0.21)	.15
CMAI†	29‐203 ^hours^	0.96 (−5.58, 3.65)	.68	0.68 (− 2.72, 4.08)	.70
DQI	−0.103‐1 ^a^	0.06 (−0.03, 0.16)	.19	0.07 (−0.02, 0.16)	.13
EQ‐5D + C	max. 1 ^a^	0.04 (−0.69, 0.15)	.46	0.03 (−0.05, 0.10)	.48
NPI†	0‐144 ^i^	0.74 (4.99, 3.50)	.73	−0.71 (−3.15, 4.49)	.73

*Note:* † based on imputed data.

*Note:* Higher scores indicate a: better quality of life; b: higher burden; c: better health; d: fewer depressive symptoms; e: more anxiety symptoms; f: longer perseverance time; g: more independency; h: more agitation; i: more symptoms.

*Note:* B represents the beta coefficient of the adjusted mean difference with the 95% confidence interval (95%CI) between parentheses.

### 3.4 Secondary outcomes in the caregiver

Secondary outcomes related to QoL and experienced burden also showed non‐significant effects: the aMDs for the Carer QoL VAS, EQ 5D‐3L, and Self‐rated Burden Scale were 0.16 (95%CI −0.38 to 0.70), 0.06 (95%CI −0.02 to 0.13), and 0.12 (95%CI −0.53 to 0.78), respectively. Effects on experienced health assessed by the RAND‐36‐SF are represented in nine subscales, and we found significant improvement in the “role limitations due to physical functioning” (aMD, 13.04; 95%CI, 3.15 to22.93) and “role limitations due to emotional functioning” (aMD, 13.52; 95%CI, 3.76 to 23.28). There was also a positive effect on experienced pain (aMD, 9.43; 95%CI, 1.00 to 17.86). However, there were no significant changes for other RAND‐36‐SF outcomes, with aMDs of 1.86 (95%CI −5.40 to 9.13) for physical functioning, 4.58 (95%CI −2.52 to 11.68) for general health perception, 2.57 (95%CI −6.69 to 11.83) for mental health, 1.52 (95%CI −5.34 to 8.39) for health change, 5.44 (95%CI −3.08 to 13.97) for social functioning, and 2.76 (95%CI −3.08 to 8.59) for vitality. Depressive and anxiety symptoms were also unchanged, with aMDs of −2.45 (95%CI −5.67 to 0.77) on the CES‐D and 0.46 (95%CI −0.21 to 1.13) on the HADS‐A, respectively. Of note, the perseverance time did not change significantly (aMD, 0.13; 95%CI, −0.27 to 0.52).

### 3.5 Secondary outcomes in the PwD

The QoL of PwD assessed by the DQI and the EQ‐5D + C showed no significant changes, with aMDs of 0.07 (95%CI, −0.02 to 0.16) and 0.03 (95%CI, −0.05 to 0.10), respectively. There were no differences between the intervention and control groups in agitation assessed by the CMAI (aMD, 0.68; 95%CI, −2.72 to 4.08) or in neuropsychiatric symptoms assessed by the NPI (aMD, −0.71; 95%CI, −3.15 to 4.49). There were also no significant differences in overall psychotropic use or in specific use of antipsychotics, antidepressants, hypnotics, and anxiolytics between the groups at baseline.

### 3.6 Subgroup analyses

Subgroup analyses were performed by sex, age, and educational level to compare differences between caregivers in the intervention and control groups for care‐related QoL (Table 3). Men in the intervention group showed a significantly better QoL (aMD, 10.16; 95%CI 1.05‐19.28), but women did not show a significant improvement (aMD, 2.85; 95%CI −5.17 to 10.87). In addition, outcomes were not significantly different in either the younger (aMD, 4.79; 95%CI, −6.07 to 15.65) or older (aMD, 7.23; 95%CI, −0.67 to 15.12) subgroups. Finally, the intervention resulted in a significant improvement in QoL in the less‐educated subgroup (aMD, 16.87; 95%CI, 7.72‐26.01), but only a non‐significant increase in QoL in the more highly educated subgroup (aMD, 2.89; 95%CI, −4.10 to 9.89).

**TABLE 3:** Subgroup analysis: primary outcome for caregivers (CarerQol 7D) after 3 months; B represents the beta coefficient of the adjusted mean difference with the 95% confidence interval (95%CI) between parentheses; n: number of participants at follow‐up at 3 months

	B (95%CI)	*P*
Women n = 60	2.85 (−5.17, 10.87)	.479
Men n = 21	10.16 (1.05, 19.28)	.031
CAREGIVER		
Young <72.8 n = 32	4.79 (−6.07, 15.65)	.374
Old >72.8 n = 49	7.23 (−0.67, 15.12)	.072
PERSON WITH DEMENTIA		
Lower education n = 20	16.87 (7.72 26.01)	.001
Higher education n = 57	2.89 (−4.10, 9.89)	.410

### 3.7 Qualitative outcomes

Of the 59 couples who participated in the intervention, 38 attended either one or both follow‐up meetings. These meetings were semi‐structured and were free to differ in the topics discussed. In the appendix an overview can be found of the caregivers' comments on the benefit experienced, the organization, and the areas for potential improvement. An important issue was the notion of improved general knowledge on dementia and the effect this had on their feelings (eg, greater acceptance) and behavior (eg, preparing for the future). Greater acceptance and improved coping were reported to result in less psychological stress and fewer negative feelings. Realization of the importance of taking care of oneself and receiving information about community services and facilities from professionals and other caregivers had stimulated caregivers to arrange to receive more support. Participants reported that they had implemented the practical knowledge acquired, that they had learned new or better skills to manage behavioral problems in PwD, and that they had begun to make plans. It should be noted that caregivers gave conflicting reports about their experiences during the intervention week, with some experiencing the week as fun and relaxing, some finding it (too) strenuous. Some feeling that they had not received enough information from the dietician (men), and some feeling that they had learned nothing new (women).

## 4 DISCUSSION

The More at Home with Dementia intervention had no significant effect on care‐related QoL, the primary study outcome. However, it did produce significant positive effects on role limitations due to physical and emotional functioning, two key secondary outcomes assessed by the RAND‐36‐SF. These latter subscales comprise two essential identical questions that: (a) “were you limited in the time you could spend on work or other activities?”; and (b) “were you limited in achieving goals?” It may have been the responses to these questions that produced the positive outcomes in each subscale. The qualitative outcomes presented in the Appendix S1 help us to understand the reasons for these positive effects on functional and emotional limitations. Many comments indicated that caregivers had gained better coping skills, which in turn, had led to them feeling less restricted. Improved coping abilities may also have accounted for the positive effect on the experience of pain, which is strongly related to psychosocial factors.

Apart from the effects on functional and emotional limitations, the main themes of the qualitative outcomes showed only limited or no agreement with the questions used in the instruments. We also noted that there was overlap with the needs of caregivers reported in a review of the psychological impact of caregiving^13^ and the results of the European Artifcare Study.^12^ The qualitative outcomes further supported the positive effects on self‐reported needs in the “Going to stay at Home” program on which this intervention was based.^21^ Moreover, the findings emphasize the importance of using a mixed‐methods approach when considering psychosocial interventions if we are to avoid the limitations of randomized controlled trials that rely solely on quantitative data.

The subgroup analyses for the primary outcome revealed that only men who received the intervention had a significantly higher care‐related QoL compared with the control group. Earlier research showed that female caregivers experience more depressive symptoms and higher burden compared to men,^6^, ^7^, ^44^ and our results suggest that men benefit more from the intervention we used. In addition, participants with relatively lower education had significantly higher QoL after the intervention compared with those receiving care as usual. This contrasted with the findings for more highly educated caregivers who may be better able to solve problems without help (eg, searching for information on the internet), and as such, may gain relatively less benefit from our intervention. This outcome may be relevant because professionals tend to recommend these types of intervention to caregivers who are more highly educated, when in fact less well‐educated groups could be more likely to benefit. Although these outcomes suggest a need for clinicians to change their referring practices, we must await further research because the small numbers in each subgroup requires that we consider any conclusion with caution.

The challenges faced by informal caregivers of PwD can easily be underestimated when discussing psychosocial interventions. Most interventions are effective to some extent, and these effects may last for varying periods, but it is undeniable that caring for a PwD is a long‐term commitment for which people will encounter different challenges at different phases. We posit that any single intervention during this process has the potential to relieve symptoms and provide the caregiver with useful knowledge and skills. However, it is unlikely that a single short‐term intervention will suffice for the needs of all caregivers in all situations, or indeed, that the positive effects will be indelible. Such an intervention should cover a longer period when possible, as was done in the New York University caregiver intervention.^45^ Likewise, the meetings after 3 and 6 months we organized in our study might have served as a support group and be continued as long as necessary. Also, we believe that multicomponent interventions like More at Home with Dementia could be enhanced by supplementing it with a personalized continuous care plan that can be adjusted as the support needs of a caregiver change,^15^ where the optimal timing of these multicomponent interventions in the trajectory of dementia should be subject of future research.

### 4.1 Strengths and limitations

A strength of this study was that qualitative and quantitative data were collected, making it possible to explain the quantitative findings with comments given by participating caregivers. Data were also collected from both the caregivers and the PwD to help place the results in context, and the research process was transparent and described in a process evaluation article. Finally, the time between the intervention and the follow‐up assessment was 3 months. This is an important strength because we wanted to assess the long‐term effects that we considered clinically more relevant. Most studies to date have performed follow‐up directly or shortly after an intervention.

This study also has some limitations. First, we included fewer participants than was calculated to be necessary in the sample size estimate, and this dropout was not evenly distributed between the study groups. This might explain why almost all outcomes non‐significantly favored the intervention, as was expected based on earlier research findings. Also, although subgroup analyses revealed positive effects on care‐related QoL for caregivers who were male or educated to a lower level, the small sample sizes and multiple testing in these groups preclude drawing meaningful conclusions. The lack of power will necessitate replication through further research. Second, the reasons for dropout differed between the intervention and control groups, with some responses indicating that participants left the control group because they did not want to complete questionnaires without receiving the intervention. Lack of further data on these participants meant that we could not estimate the effect of this selective dropout on the outcomes.

### 4.2 Conclusions

The multicomponent training program, More at Home with Dementia, has no significant effect on care‐related QoL, but does meet the needs of caregivers living with PwD. After the intervention, caregivers may experience fewer role limitations due to physical and emotional function, and may suffer less pain. We believe that there is growing evidence that such interventions have positive effects on the lives of caregivers, and indirectly PwD, but that single short‐term programs cannot relieve all problems for all people indefinitely. Due to the changing nature of dementia and its care requirements, any multicomponent training program for caregivers and PwD should ideally form part of a continuous and personalized care plan for that dyad.

## CONFLICT OF INTEREST

The authors declare that they have no competing interests. However, there were two commercial sources of Funding (THEIA foundation of Zilveren Kruis Health insurance and Laurens Care Centers, Rotterdam, the Netherlands). The commercial funders had no role in the design, conduct, analysis and final publication decisions of the study.

## Supporting information


**Appendix** **S1.** Qualitative analysis: comments by caregivers during follow‐up meetings ordered by categories and themes.

## Data Availability

The data that support the findings of this study are available on request from the corresponding author. The data are not publicly available due to privacy or ethical restrictions.
