# Supplementary material for: Effects of caregiver dementia training in caregiver‐patient dyads: A randomized controlled study
Source: Int J Geriatr Psychiatry. 2020 Jul 22;35(11):1376–84. doi: 10.1002/gps.5378 (PMC7689696; doi:10.1002/gps.5378)
Supplement: Supplementary file 1 — Appendix S1. Qualitative analysis: comments by caregivers during follow‐up meetings ordered by categories and themes. [file GPS-35-1376-s001.docx]

**Appendix**

**Qualitative analysis: comments by caregivers during follow-up meetings ordered by categories and themes**

| **General knowledge about dementia and caregiving** |
| --- |
| Improved knowledge of dementia (aids acceptance) |
| Learned to ask for help (including the importance of doing so) |
| Learned the importance of taking care of oneself (doing pleasant things, taking time for yourself, taking better care of own health) |
| Realized that preparing for the future is important |
| Were stimulated to start to make adaptations and to use facilities |
| **Changes in psychological stress and emotional well being** |
| Less feelings of stress, feels calmer |
| Better coping in with the problems that come with the dementia of his or her partner in general |
| Became more laconic |
| Implementing his or her own solutions and greater self esteem |
| Less irritated by the behavioral problems of his or her partner |
| More acceptance on the situation |
| Learned to control themselves |
| Learned not to feel guilty about feelings of irritation |
| **Changes in the organization of support** |
| Asked for professional help for own psychological problems |
| Learned that social activities are important for his or her partner and organized these |
| Arranged practical help for household chores |
| Arranged help from a case manager on dementia |
| Started or increased day care |
| Started to participate in a support group for caregivers |
| **Improved coping with changes that arise with dementia in a partner** |
| Started therapeutic activities, aiming to let his or her partner do as much as possible themselves |
| Learned to ask one thing at a time and to use short sentences |
| Used the workshop of the registered nurse on how to help to dress his or her partner |
| Started to use available facilities or services (e.g., transport) |
| Started to use a personal alarm |
| Realized that physical exercise and going outdoors is important and put these into practice |
| Learned to be patient and not to hurry |
| Adjusted his or her home based on the advice of the occupational therapist |
| Approved of the workshop by the speech therapist on swallowing and paid the topic more attention |
| Realized and put into practice that his or her partner can still learn certain things |
| Put advice on home automation into practice (e.g., night lights with motion sensors) |
| Used information on color contrast (e.g., setting the table or the floor in the bathroom) |
| Bought a simpler cell phone for his or her partner |
| **Changes in coping skills concerning the behavioral problems of their partners** |
| Better coping with his or her partner’s resistant behavior |
| Less inclined to try to control his or her partner |
| Learned to cope with a partner who doesn’t recognize his house while being at home |
| Stopped correcting the mistakes of partner |
| **Planning for the future** |
| implemented advanced care decisions (e.g., a living will) |
| Orientation on the need for nursing homes |
| Began to organize their Organized financial affairs |
| **Positive experiences during the course** |
| Appreciated being taken care of for one week |
| Caregivers Felt like it was a holiday feeling rather than a burden |
| Being able to sharing problems that come with having a partner with dementia was appreciated (e.g., recognizing each other’s stories) |
| Observing how other caregivers deal with their partners was helpful and their experience could be used |
| Appreciated the group feeling |
| Partners reported that they had experienced a holiday feeling and had a fun week |
| **Negative experiences during the course** |
| There was overlap between workshops |
| The course was long and intensive, which could feel tiresome and burdensome |
| The program for the person with dementia was not sufficient |
| Some information seemed superfluous (e.g., sense that when you take care of your partner for a long time you have developed your own strategies) |
| Listening to the same stories from each caregiver, but with different professionals, was tedious |
| There was not enough spare time allocated during the week |
| **Areas for improvement** |
| More information about community services |
| More information about holidays for persons with dementia |
| More attention for individual problems (e.g., a private session with the psychologist) |
| Missed attention for the grief and anger that is aroused by having a partner with dementia |
| More info about GPS systems |
| More information on healthy food and its connection with dementia (men) |
| Information on food was not necessary (women) |
| More attention on the problems that come with the changed relationship when a partner has dementia |
| More practical advice about how to improve the activity level of activate your partner |
| More information about the physical changes that come with dementia (e.g., apraxia) |
| More information about how to cope with a partner who denies his or her cognitive problems |
| Having two people carry out the program for their partners, giving more possibilities to fine tune those activities |
| **Syllabus** |
| The syllabus was used to read about issues after the intervention |
| The syllabus could make it possible to apply the information in a later stage of the dementia |
| The syllabus was useful to help inform people at home about the course content |
